# isa4j: a scalable Java library for creating ISA-Tab metadata

**DOI:** 10.12688/f1000research.27188.1

**Published:** 2020-12-03

**Authors:** Dennis Psaroudakis, Feng Liu, Patrick König, Uwe Scholz, Astrid Junker, Matthias Lange, Daniel Arend

**Affiliations:** 1 Leibniz Institute of Plant Genetics and Crop Plant Research (IPK) Gatersleben, Seeland, 06466, Germany; 2 Hochschule Mittweida, University of Applied Sciences, Mittweida, 09648, Germany

**Keywords:** ISA-Tab, FAIR data, reproducible research, metadata, Java, object-oriented programming, framework

## Abstract

Experimental data is only useful to other researchers if it is findable, accessible, interoperable, and reusable (FAIR). The ISA-Tab framework enables scientists to publish metadata about their experiments in a plain text, machine-readable format that aims to confer that interoperability and reusability. A Python software package (isatools) is currently being developed to programmatically produce these metadata files. For Java-based environments, there is no equivalent solution yet. While the isatools package provides a lot of flexibility and a wealth of different features for the Python ecosystem, a package for JVM-based applications might offer the speed and scalability needed for writing very large ISA-Tab files, making the ISA framework available in an even wider range of situations and environments. Here we present a light-weight and scalable Java library (isa4j) for generating metadata files in the ISA-Tab format, which elegantly integrates into existing JVM applications and especially shines at generating very large files. It is modeled after the ISA core specifications and designed in keeping with isatools conventions, making it consistent and intuitive to use for the community.

isa4j is implemented in Java (JDK11+) and freely available under the terms of the MIT license from the Central Maven Repository ( https://mvnrepository.com/artifact/de.ipk-gatersleben/isa4j). The source code, detailed documentation, usage examples and performance evaluations can be found at https://github.com/IPK-BIT/isa4j.

## Introduction

In recent years, the question of how to publish research data has increasingly come into the limelight of discussions among scholars, funders, and publishers ^1^. Wilkinson *et al.* ^2^ establish a set of principles to ensure that data are shared in a way that is useful to the community and worthwhile for data producers: Data should be findable, accessible, interoperable, and reusable (FAIR) – not only by humans but also by computers. In some scientific fields, there are well-curated, consistent, and strongly integrated databases that provide easy access for both humans and machines, such as Genbank and UniProt for nucleotide and protein sequences ^3, 4^. Other areas, like plant phenotyping, do not yet have central databases or established file formats and things become especially difficult when data from different domains need to be published in conjunction. The Investigation-Study-Assay (ISA) framework and the corresponding ISA-Tab file format ^5^ provide a clearly defined, machine-readable, and extensible structure for explanatory metadata that bundles common elements while keeping data in separate files using appropriate formats. Several communities have already created specific standards (such as MIAPPE ^6^ or MIAME ^7^) and infrastructure ^8^ based on the ISA framework. Furthermore, tools have been developed for validating, converting, and manually crafting ISA-Tab metadata ^7, 9, 10^. However, given the ever-increasing volume of research data generated in high-throughput experiments, the manual creation of metadata is simply not feasible in many situations. A Python package called isatools for programmatically generating ISA-Tab metadata is currently under development ( https://isatools.readthedocs.io) featuring methods to parse, validate, build, and convert ISA files. It also offers a feature to create sample collection and assay run templates according to a specified experimental design which can be useful when planning an experiment. Building ISA-Tab files, isatools provides great flexibility and ease of use: users can create and connect ISA objects in arbitrary order and degree of detail and isatools automatically determines the appropriate formatting when the ISA-Tab text is rendered.

**Figure 1.**
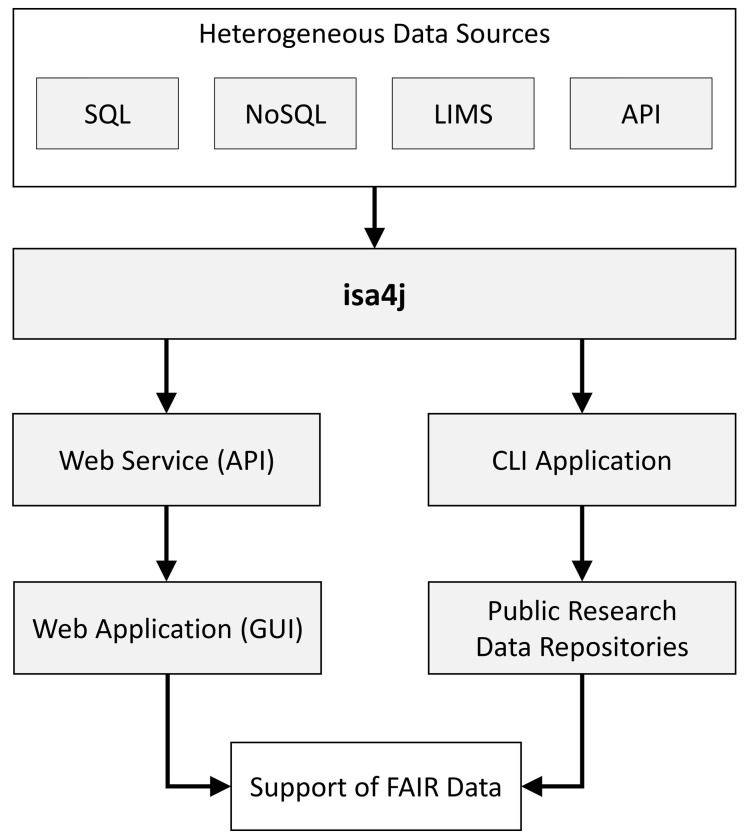
Exemplary integration of isa4j into different application scenarios for supporting the FAIR data sharing paradigm. Heterogeneous data sources like SQL and NoSQL databases, laboratory information management systems (LIMS) and application programming interfaces (API) that store data and metadata of scientific experiments can be fed into isa4j to integrate and transform this data to output complying with the ISA specifications: The isa4j library can, for example, be embedded in command line interface (CLI) applications to create ISA-Tab files in a batch processing manner. It may also be embedded in web services to create ISA-Tab files on the fly via an API based on specific user requirements. ISA-Tab files created with CLI applications could be uploaded to public research data repositories for long-term storage and web applications as graphical user interfaces would allow low-barrier interactive access to experimental data. Both examples demonstrate how isa4j can be used for FAIR data sharing.

Naturally, this flexibility requires isatools to keep the whole object structure in memory and resolve the optimal path through the object chain when the content is serialized. This can notably impact performance when describing large and complex studies including a high number of replicates and attributes, as for instance required by the MIAPPE standard for plant phenotyping experiments. This could make it challenging to use isatools in interactive and time-sensitive applications. Additionally, in the majority of cases, the desired file structure is already clear beforehand based on such community standards or your own decision of what needs to be documented, so this flexibility is often not needed. We therefore set out to develop a solution that focuses on high performance and scalability, and which would integrate well into JVM-based data publishing ecosystems. The library, called isa4j, addresses these goals by providing interfaces for exporting ISA-formatted metadata not only to files, but also to any data stream provided by the application (e.g. a HTTP response stream in a web application) and using an iterative approach for creating ISA-Tab files: Instead of loading all records into memory and writing them in one go, an output stream is opened, a single record is created, flushed out into the stream, and then immediately dropped again from memory. This guarantees memory usage to remain constant so that isa4j imposes no limit on the size of the generated metadata and is able to process datasets too big to fit into memory. The output stream can also be picked up by the application and piped into further processing steps, such as calculating checksums or compressing the ISA-Tab content. In exchange, the user needs to structure rows consistently as headers cannot be modified once they are written. The schema in Figure 1 shows the exemplary integration of isa4j into different application scenarios for supporting the FAIR data sharing paradigm. In this article, we explain how isa4j can be used to generate ISA-Tab metadata and compare it to isatools in performance and scalability regarding both quantity and complexity of ISA-Tab entries.

## Methods

### Implementation

isa4j is implemented in Java (JDK11+) and can therefore also be used with other JVM-based languages like Groovy or Kotlin. It uses the Gradle Build Tool ( https://gradle.org) to resolve dependencies and create arti-facts. Logging is realized via the framework-agnostic SLF4J library ( http://www.slf4j.org/) so that isa4j works with a variety of logging libraries. The object-oriented Java class structure is modelled according to the published ISA specifications ( https://isa-specs.readthedocs.io) to make isa4j intuitive to use and keep consistency with other ISA applications. The Ontology and OntologyAnnotation classes allow linking characteristics, units, and other metadata to established vocabularies such as those collected by the OBO Foundry ^11^.

### Operation

isa4j is not an application itself but a software library providing methods for generating ISA-Tab metadata in JVM-based applications or scripts. As a result, operation requires at least a basic level of coding skills in Java or another JVM-based language. When using a build tool like Maven or Gradle, isa4j can simply be added as a dependency to be downloaded from the Central Maven Repository ( https://mvnrepository.com/artifact/de.ipk-gatersleben/isa4j). Otherwise, the JAR file can be downloaded from there and manually included in the class path. To use isa4j’s logging feature, one of the SLF4J bindings needs to be included the same way ( http://www.slf4j.org/manual.html). 

You can then import isa4j classes and start building Investigation, Study, and Assay files. For examples and details on the code interface itself, please consult the current project page ( https://github.com/IPK-BIT/isa4j) as things may change in future versions and we do not want to confuse you with potentially outdated information.

### Scalability evaluation

Scalability of isa4j was assessed and compared to the Python isatools API in two dimensions: number of entries and complexity of entries.

At the simplest complexity level ( *Minimal*), Study file rows consisted only of a Source connected to a Sample through a Process, and that Sample connected to a DataFile through another Process in the Assay File, with no Characteristics, Comments, or other additional information (6 columns in total). At the second degree of complexity ( *Reduced*), a Characteristic was added to the Sample in the Study File, and the Assay File was expanded to include an intermediary Material Object (11 columns). The third and final level of complexity ( *Real World*) was modelled after the MIAPPE v1.1 compliant real-world metadata published for a plant phenotyping experiment ( https://doi.org/10.5447/IPK/2020/3, 119 columns). Exemplary ISA-Tab output for each of the three complexity levels can be found at https://ipk-bit.github.io/isa4j/scalability-evaluation.html#complexity-levels. 

For each complexity level, CPU execution time was measured for writing a number of *n* rows in Study and Assay File each, starting at 1 and increasing in multiplicative steps up to a million rows. Every combination of complexity level and number of rows was measured for 5 consecutive runs in isatools and 15 runs for isa4j (here results varied more) after a warm-up of writing 100 *Real World* complexity rows. Additionally, memory usage was measured for realistic complexity in 5 separate runs after CPU execution time measurements. 

All evaluations were carried out on a Linux server with two Intel Xeon E5-2697 v2 CPUs running at 2.70 GHz, 256 GB DDR3 RAM running at 1600 MHz and CentOS 7.8.2003. isatools was evaluated under Python 3.7.3 [Clang 11.0.0 (clang-1100.0.33.16)] using isatools version 0.11 and memory-profiler version 0.57 for measuring RAM usage. isa4j was evaluated under AdoptOpenJDK 11.0.5. For both libraries, a memory consumption baseline was calculated after the warm-up runs and an additional Garbage Collector invocation. This baseline consumption was subtracted from all subsequent memory consumption values as we wanted to measure purely the memory consumed by the ISA-Tab content, not libraries and other periphery ^1^. The actual code generating the files and measuring time and memory usage for Python isatools ^2^ and isa4j ^3^ can be found on the isa4j GitHub repository.

## Results

**Figure 2.**
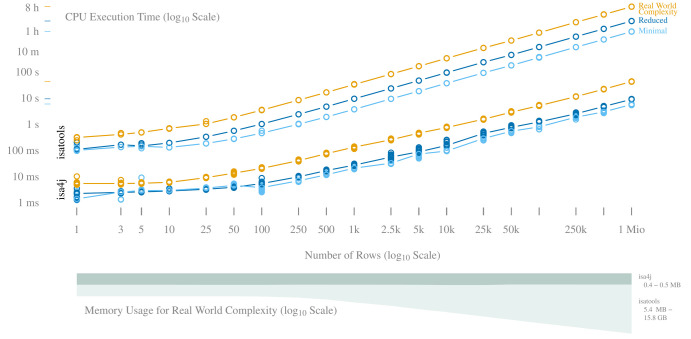
Performance comparison of isa4j and isatools. Top: CPU execution time at 3 different row complexity levels; Real World complexity was modelled after MIAPPE v1.1 compliant ISA-Tab metadata generated for a plant phenotyping experiment. Small colored lines on the left mark the highest point of each curve to help estimate the maximum value. Bottom: Memory consumption for isa4j and isatools along the same x-axis.

Figure 2 shows the performance of both libraries at increasing file size for three different levels of complexity. isa4j consistently takes up less CPU execution time than isatools for all tested scenarios, reducing the time required for writing 1 million rows of Real World complexity from 8.6 hours to 43 seconds.

The emphasis on being useful especially in large-scale datasets is further amplified by isa4j’s memory usage stability: While there is no notable increase for either library up to a volume of 25 rows, starting at about 250 rows, isatools memory consumption increases linearly with the number of rows being formatted, resulting in a maximum consumption of 15.8 GB for one million rows. isa4j memory consumption remains stable at about 0.5 MB independently of the number of rows written, demonstrating that the iterative technique of formatting and writing the rows had the desired effect.

### Use Case: BRIDGE Web Portal

**Figure 3.**
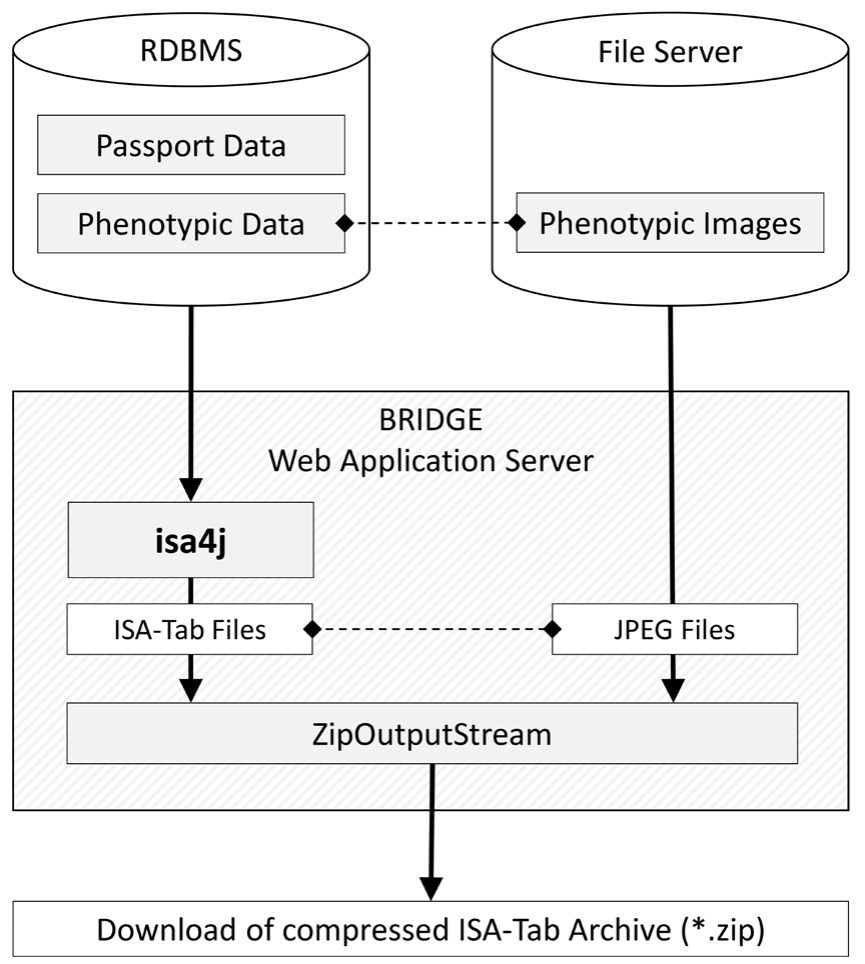
BRIDGE process flow. The BRIDGE web application uses isa4j to create ISA-Tab files from experimental data stored in a relational database management system (RDBMS). The data consists of passport data ^14^ describing the basic characteristics of a germplasm sample (such as accession number and location of origin) on the one hand, and of phenotypic data, which systematically describe phenotypic characteristics of the individual germplasm samples, on the other. Phenotypic images that are stored as binary files on a separate file server are linked from the ISA-Tab files and are included in the final ISA-Tab ZIP archive.

We have integrated isa4j into the BRIDGE portal, which is a visual analytics and data warehouse web application hosting data of 22621 genotyped and 9527 phenotyped germplasm samples of barley ( *Hordeum vulgare* L.) ^12^. The underlying data was derived from the study of Milner *et al.* ^13^. isa4j was integrated to allow the MIAPPE-compliant ^16^ export of customized subsets of phenotypic data of germplasm samples together with the corresponding passport data ^14^ in the ISA-Tab format. These subsets can be derived from germplasm selections identified by the user during exploratory data analysis. In the ISA-Tab export dialog, the user can choose whether the associated plant images should be physically contained as files in the resulting ZIP file or whether they should only be linked as URLs to a version of the images available online. Due to the support of streaming in isa4j, the phenotypic data export module of BRIDGE is able to export large ZIP archives of several gigabytes with low main memory consumption of the web server. Another advantage over non-streaming approaches is that the download can start without delay and that no temporary files have to be created on the server. The process flow concept is shown in Figure 3.

## Discussion

We have created a library for programmatically generating ISA-Tab metadata files in JVM-based environments and shown that it is considerably more performant and scalable than the existing Python based solution. It has been integrated into a large-scale data warehouse web software to validate practical feasibility and provide an example of how the library could help make ISA-Tab metadata available in time-sensitive applications.

CPU execution time appears to have a roughly linear relationship with the number of rows being written at *n* > 250 but this is only valid as long as isatools memory consumption does not surpass what the system can provide. Exceeding that, additional time for swapping from and to the hard disk will be required. There may also be further non-linear effects due to optimization steps, such as the compilation to native machine code some JVMs perform for frequently used code parts. Lastly, exact CPU time requirements will naturally depend on the specific system in use but the overall relationships and proportions shown here should hold true for all situations.

## Conclusions

The presented isa4j library provides a simple interface to create and export ISA-Tab metadata and can be seamlessly integrated into existing JVM-based pipelines, desktop tools or web applications. isa4j is less flexible than the Python-based isatools as it does not allow one to change the file structure after streaming has started, but the desired ISA-Tab configuration is often known beforehand, making this a peripheral limitation. In exchange, isa4j provides significantly better performance, especially for large datasets. We hope that this library will make the ISA framework available to an even wider audience and range of situations and help make published research data more interoperable and reusable for others. As a next step, we are going to begin developing a specialized isa4j extension for plant phenotyping experiments, isa4j-miappe, intended to make it even easier for researchers in the field to ensure their metadata comply with the community standard. If you would like to contribute or develop an isa4j extension for your own community, please feel free to get in touch with us.

## Data availability

Raw performance measurement data can be found at https://raw.githubusercontent.com/IPK-BIT/isa4j/master/docs/performance_data.csv (archived: Zenodo, IPK-BIT/isa4j: isa4j-1.0.4, http://doi.org/10.5281/zenodo.4275168 ^15^).

## Software availability

Software available from: https://mvnrepository.com/artifact/de.ipk-gatersleben/isa4j

Source code available from: https://github.com/IPK-BIT/isa4j Archived source code as at time of publication: http://doi.org/10.5281/zenodo.4275168 ^15^

License: MIT

## Footnotes

^1^ Baseline memory consumption was approximately 100 MB for isatools and 11 MB for isa4j.

^2^ https://github.com/IPK-BIT/isa4j/blob/master/src/test/resources/de/ipk_gatersleben/bit/bi/isa4j/performanceTests/isatools_performance_test.py

^3^ https://github.com/IPK-BIT/isa4j/blob/master/src/test/java/de/ipk_gatersleben/bit/bi/isa4j/performanceTests/PerformanceTester.java
