# Supplementary material for: isa4j: a scalable Java library for creating ISA-Tab metadata
Source: F1000Res. 2020 Dec 3;9:ELIXIR-1388. [Version 1] doi: 10.12688/f1000research.27188.1 (PMC7941097; doi:10.12688/f1000research.27188.1)
Supplement: Peer review history

# Peer review: Reviewer response for version 1

**DOI:** 10.5256/f1000research.30037.r79165

**Published:** 2021-03-08

**Authors:** Nils Hoffmann

**Affiliations:** 1 Center for Biotechnology (CeBiTec), Bielefeld University, Bielefeld, 33594, Germany

The authors describe a JAVA-based implementation of the Investigation-Study-Assay (ISA) framework for the structured description of biological experiments, their protocols and their results. They position their implementation as a complement to the existing Python-based implementation that uses a complete in-memory model of the ISA data structures before writing them to the actual output files. For large studies, e.g. for whole populations or large cohorts, this can mean that memory and CPU requirements are very demanding.

 Thus, the authors implemented their JAVA library to write out lines as they arrive, requiring that the user fix their data format description before starting to write out to the final files. Therefore, their implementation's memory complexity scales constant with the number of rows to be written, as each row can be created ad-hoc and then written out to the target file. In order to underline this advantage over the Python-based library, the authors created different benchmarks, illustrating the memory and CPU time usage of each library for a collection of different study designs with increasing levels of complexity, highlighting the significant speed and memory advantage of their implementation.

 Finally, the authors demonstrate the practical feasibility of their library through integration into the BRIDGE web portal where they employ isa4j to generate ISA-tab files on the fly for the studies stored in BRIDGE.

 The support for ISA-tab in programming languages other than Python, especially with a focus on performance, is a timely and needed addition. For JAVA, the graphical client ISACreator was previously developed, but has not seen any significant updates throughout the last few years. Specifically, where neither the flexibility of the Python-based ISA tools nor a graphical user interface for a predefined ISA format are required, the isa4j library can be a valuable, performant, yet still validating tool to generate ISA-tab files in many different life-sciences domain, such as metabolomics, proteomics, genomics, etc. Thus, it addresses a current need and does this in a well designed and performant way.

**Minor comments:**

- The manuscript states, that the library is available from mvnrepository.com, while the GitHub page states that it is available from Maven Central, please update the manuscript accordingly.
- The online documentation to set up a custom isa4j project should also mentions that an slf4j logging implementation needs to be on the class path, e.g. for Gradle:
  implementation 'org.slf4j:slf4j-simple:1.7.30'
- The documentation at https://ipk-bit.github.io/isa4j/getting-started/investigation-file.html in section "Add them as investigation contacts" is syntactically not correct (missing semicolons ';' ), also the method findByName does not exist in version 1.0.4 of the library (getByName does).
- I would recommend to set up a continuous integration system linked to the GitHub repository (GitHub actions, Travis, CircleCI, etc.) to make sure that the source code is buildable and automatically tested.

Are the conclusions about the tool and its performance adequately supported by the findings presented in the article?

Yes

Is the rationale for developing the new software tool clearly explained?

Yes

Is the description of the software tool technically sound?

Yes

Are sufficient details of the code, methods and analysis (if applicable) provided to allow replication of the software development and its use by others?

Yes

Is sufficient information provided to allow interpretation of the expected output datasets and any results generated using the tool?

Yes

Reviewer Expertise:

Bioinformatics, mass spectrometry of small molecules, data standardization.

I confirm that I have read this submission and believe that I have an appropriate level of expertise to confirm that it is of an acceptable scientific standard.

# Peer review: Reviewer response for version 1

**DOI:** 10.5256/f1000research.30037.r77273

**Published:** 2021-02-16

**Authors:** Massimiliano Izzo

**Affiliations:** 1 Oxford e-Research Centre, Department of Engineering Science, University of Oxford, Oxford, UK

The authors present isa4j, an optimised Java-based library to generate and serialise ISA-TAB metadata. I find particularly interesting that isa4j supports writing the ISA-metadata output on streams as well as files, as this can be very useful when building modern client-server applications. isa4j has an interesting approach of loading into memory only one row at the time, hence limiting memory consumption. The memory consumption comparison with isatools makes a good argument for using isa4j for certain large scale experiment.

 I am curious to know whether isa4j-generated ISA-TABs comply with the ISA-TAB validation rules, also with respect to the configuration files for specific assays (latest version can be found here. There are a few discrepancies with respect to the official ISA-TAB specifications: for instance, Processes cannot have names in "isa4j" and as a consequence "Assay Name" or synonymous columns are missing. Characteristic categories are treated as strings in isa4j, while are OntologyAnnotations in the ISA-API. I might have missed other, minor, discrepancies. I would suggest adding more equivalence tests with existing datasets to align more this library with isatools.

 The developers don’t seem to have put the tests into continuous integration; I think it would be worth doing so.

 In any case, I think isa4j is a useful tool with strong performance, that will be very helpful to produce ISA-TAB metadata from a variety of large-scale experiments with noteworthy performances and low resources consumption.

Are the conclusions about the tool and its performance adequately supported by the findings presented in the article?

Yes

Is the rationale for developing the new software tool clearly explained?

Yes

Is the description of the software tool technically sound?

Yes

Are sufficient details of the code, methods and analysis (if applicable) provided to allow replication of the software development and its use by others?

Yes

Is sufficient information provided to allow interpretation of the expected output datasets and any results generated using the tool?

Yes

Reviewer Expertise:

Applied Computer Science, Software Engineering

I confirm that I have read this submission and believe that I have an appropriate level of expertise to confirm that it is of an acceptable scientific standard.
